# Nano-Engineering Solutions for Dental Implant Applications

**DOI:** 10.3390/nano12020272

**Published:** 2022-01-15

**Authors:** Karan Gulati

**Affiliations:** School of Dentistry, The University of Queensland, Herston, QLD 4006, Australia; k.gulati@uq.edu.au

This Special Issue of *Nanomaterials* explores the recent advances and trends with respect to nano-engineered strategies towards dental implant applications. A dental implant microenvironment is complex, and an implantation surgery results in a local trauma [1]. Further, exacerbated by the ongoing patient conditions (age, osteoporosis, diabetes or smoking), long-term dental implant success may be compromised due to inappropriate integration (both soft-tissue and osseo-integration), inflammation and bacterial infection [2,3]. As a result, surface modification of dental implants to fabricate desirable topographical and chemical features towards enhancing osseo- and soft-tissue integration (STI), has been well documented [4]. Various physical, chemical and biological modifications have been investigated across the macro-, micro- and nano-scales to find the most optimum dental implant surface features [5].

The goal of this Special Issue is to shine light on the recent nano-engineering advances that revolutionize the dental implant technology, with a focus on the next generation of implants capable of providing maximum local therapy to drastically reduce implant failures. This Special Issue will inform the readers of the latest nano-engineering developments in the domain of dental implants, aiming to bridge the gap between research and clinical translation, from lab to clinics. This Special Issue contains a blend of eight original research, communication-style research and review papers from leading scientists across the world with expertise in nano-engineered dental implant technology.

Titanium (Ti) is the most popular choice for the fabrication of dental implants and hence several articles were focussed on surface modification of Ti-based dental implants to augment their bioactivity or therapeutic potential, as reviewed by Zhang et al. [6]. The review summarizes key progress, challenges and research gaps relating to nano-engineered dental implants, spanning across the use of nano-engineered Ti and therapeutic nanoparticle (NP) modification of Ti dental implants. Similarly, the importance of nanoscale surface modification with respect to achieving desirable microbial decontamination and antibacterial efficacy is reviewed by Hosseinpour et al. [7]. While metallic and non-metallic NPs have shown great promise in both bioactivity and antimicrobial functions, natural micro-/nano-particles such as extracellular vesicles (EVs, membrane bound lipid particles secreted by all cell types) possess considerable therapeutic potential. Hua et al. reviewed the current status of periodontal and dental pulp cell derived small EVs towards anti-inflammatory, osteo/odontogenic, angiogenic and immunomodulatory functions, suitable as effective therapeutic molecules for alleviating dental implant challenges [8]. Next, Alali et al. investigated the soft-tissue integration and antibacterial performance of Lithium (Li)-doped Ti implants [9]. Briefly, chemically modified Ti doped with Li presented an extracellular matrix (ECM) mimicking nanowire network that enhanced collagen-I and fibronectin gene expression (of cultured human gingival fibroblasts) and reduced bacterial metabolic activity (of *Staphylococcus aureus*), confirming the suitability for dental implant applications.

Electrochemical anodization of Ti-based dental implants has been utilized to fabricate controlled titania (TiO_2_)-based nanotopographies including nanotubes or nanopores to augment cellular functions towards soft- and osseo-integration and enable loading and release of potent therapeutics (antibiotics or proteins) [10,11]. Briefly, anodization involves immersion of metal electrode/implant (anode) and a counter metal electrode (cathode) in an appropriate electrolyte containing water and fluoride ions and supply of optimized voltage and current, which facilitates self-ordering of various metal-oxide nanostructures on the implant (anode) surface [12]. It is known that nanoscale implant surface can influence blood coagulation that can modulate cellular functions and early osseointegration. Further, long non-coding (Lnc) RNAs regulate various processes within the skeletal system, however, the interdependence between LncRNAs (derives from clot cells) and osseointegration remains unexplored. Bai et al. bridged this research gap and investigated the correlation between LncRNAs and TiO_2_ nanotube (TNT) modified Ti implants towards osseointegration [13]. Briefly, the sequence analysis (detailed Gene Ontology and Kyoto Encyclopedia of Genes and Genomes pathway investigation) of LncRNAs (expressed within the clot formed) on TNTs of various diameters (15, 60 and 120 nm) indicated that implant nanotopography can influence the clot-derived LncRNAs expression profile, which dictates the *de novo* bone formation.

Besides Ti, Zirconium (Zr) or Zirconia (ZrO_2_) is emerging as a popular dental implant material choice attributed to its reduced affinity to bacterial plaque, appropriate mechanical properties, white colour and non-magnetic nature [14]. In a pioneering study, Chopra et al. reported nano-engineering of curved and micro-rough Zr surfaces via electrochemical anodization to fabricate various nanotopographies [15]. Briefly, by optimizing anodization conditions, dental implant/abutment relevant surfaces were modified with ZrO_2_ nanotubes, nanocrystals or nanopores, bringing anodization of dental implants closer to clinical translation.

Peri-implantitis is characterized by peri-implant mucosa inflammation and progressive destruction of the supporting bone attributed to biofilm formation [1,2]. Due to the high prevalence of peri-implantitis, various debridement techniques including mechanical treatment, chemical disinfection, antibiotic treatment, lasers and their combinations have been explored. Among these, the use of various lasers like erbium-doped: yttrium, aluminum and garnet (Er:YAG); and erbium, chromium-doped: yttrium, scandium, gallium and garnet (Er, Cr:YSGG) lasers have been proposed for implant debridement. Advancing this domain, Secgin-Atar et al. investigated the use of erbium lasers (Er:YAG and Er, Cr:YSGG) and mechanical methods (curette, ultrasonic device) on implant debridement (of implants lost to peri-implantitis) to obtain implant characteristics similar to virgin implants [16]. In total, 28 failed implants (4 failed implants in each group: titanium curette; ultrasonic scaler; Er:YAG very short pulse; Er:YAG short-pulse; Er:YAG long-pulse; Er, Cr:YSGG1; Er, Cr:YSGG2) were debrided for 120s and compared with two virgin implants (as controls) using SEM, EDX and profilometry characterizations. The results indicated that ultrasonic and Er:YAG long pulse groups were most effective debridement techniques.

Next, Casarrubios et al. studied the influence of Ipriflavone (IP) incorporated SiO_2_–CaO mesoporous bioactive glasse based hollow nanospheres (nanoMBGs) as an alternative to bioactive glasses for treating periodontal defects [17]. The authors reported that nanoMBG–IPs entered pre-osteoblasts and enabled their differentiation into mature osteoblast phenotype and enhanced the alkaline phosphatase activity, demonstrating the osteogenic potential of the nanoMBGs, which can be used towards periodontal augmentation.

In summary, this Special Issue in *Nanomaterials* entitled “Nano-Engineering Solutions for Dental Implant Applications” compiles a series of cutting-edge research and extensive review articles demonstrating the potential of advance nano-engineering towards fabrication of the next-generation of bioactive and therapeutic dental implants that overcome challenges associated with conventional implants, while maintaining clinical translatability. The Special Issue also informs the readers of the current challenges and future directions in this domain. The Editor would like to thank all contributing authors for the success of the Special Issue. This Special Issue would not have been of such quality without the constructive criticism of the Reviewers.
